# The magnitude of type 2 diabetes mellitus and cardiovascular disease risk factors among young adults in urban settings: a cross-sectional survey in Mwanza, Tanzania

**DOI:** 10.11604/pamj.2022.42.19.22184

**Published:** 2022-05-10

**Authors:** Evangelista Kenan Malindisa, Emmanuel Balandya, Fredirick Mashili, Shabani Iddi, Marina Njelekela

**Affiliations:** 1 Department of Physiology, Catholic University of Health and Allied Sciences, Mwanza, Tanzania,; 2 Department of Physiology, Muhimbili University of Health and Allied Sciences, Dar Es Salaam, Tanzania,; 3 Deloitte Consulting Limited, Dar Es Salaam, Tanzania

**Keywords:** Diabetes mellitus, dyslipidemia, hypertension, abdominal obesity, young adults

## Abstract

**Introduction:**

traditionally, non-communicable diseases were diseases of public health concern in developed countries. Due to economic transition, they are becoming more prevalent in low and middle-income countries. Despite the trend, little has been done in the population of young adults of developing countries. This research aimed to explore the magnitude of type 2 diabetes mellitus, hypertension, dyslipidemia, and abdominal obesity among the young adult population in an urban setting of Tanzania.

**Methods:**

the current research used a cross-sectional community-based design, involving apparently healthy young adults aged 18 to 34 years, not known to have diabetes, hypertension, or dyslipidemia. Data on socio-demographic characteristics, medical history, anthropometry, blood pressure, and lipids were obtained per standard operating procedures and analyzed using STATA 13. Association between outcome variables (type 2 diabetes mellitus, hypertension, dyslipidemia, and abdominal obesity) and predictor variables (age, sex, education level, occupation, and economic status) were assessed by logistic regression.

**Results:**

245 young adults with a median age of 21 (interquartile range [IQR]: 18-25) were recruited. Prevalence of diabetes mellitus and of impaired glucose tolerance (IGT) were 7.8% and 15.5% respectively. Abdominal obesity and dyslipidemia were present in 11.8% and 45.1% respectively. 34.3% had hypertension and the risk was significantly higher in males compared to females (OR 1.8, 95%CI 1.1, 3.1). The atherogenic coefficient was significantly associated with abdominal obesity; other atherogenic indices did not show significant associations with current disease conditions.

**Conclusion:**

alarmingly high prevalence of diabetes mellitus, impaired glucose tolerance, hypertension, abdominal obesity, and dyslipidemia were observed among young adults in Mwanza. This study highlights the need for concerted efforts for interventions targeting young adults in combating diabetes and cardiovascular disease (CVD) risk factors in Tanzania.

## Introduction

The burden of non-communicable diseases (NCDs) has increased considerably in the past three decades and accounts for 27% of overall mortality [1]. NCDs accounted for an estimated 17.7 million deaths (nearly one-third of all global deaths) in 2015, with more than two-thirds of NCD-related deaths occurring in low and middle-income countries (LMICs) [1,2]. NCDs are projected to be the leading cause of death and disability globally, estimated to account for more than 24 million deaths by 2030 [3]. NCDs not only cause poor health but also lead to high health expenditure, poor productivity, and poverty [4].

The increasing burden of NCDs in LMICs is fueled mainly by four modifiable risk factors; smoking, excessive alcohol drinking, sedentary lifestyle accompanied by physical inactivity, and unhealthy dietary habits characterized by consumption of energy-dense foods [5]. The current reported prevalence of diabetes in young adults of LMIC is as high as 11.6% [6], while that of hypertension is up to 36.9% [7,8], and up to 36% of young adults have dyslipidemia [9]. Among the reported NCDs in the young adult population, hypertension seems to be more prevalent and presents a sequel for multiple cardiovascular diseases (CVDs) [10].

In Tanzania, type 2 diabetes (T2D) and CVD risk factors in a young adult population have been observed to be rising, calling for an urgent intervention [11]. Diabetes prevalence has ranged from 1% [12] to 11% [13], and the prevalence of obesity has ranged from 6.7% to 28.7% [14], while abdominal obesity has been reported in 12.96% of young adults [15]. The high prevalence of T2D and CVDs in the young adult population calls for urgent interventions to protect the future generation [11]. The intercessions are of great value in LMICs like Tanzania, where the costs of healthcare are high and out of reach by the majority [11]. Overwhelming financial burden calls for an expanded venture in both preventive and curative angles of NCDs in LMIC [4].

Lifestyle adjustments aiming at reducing the energy intake and increasing energy expenditure such as physical workouts, even of a modest degree, are protective against a vast majority of NCDs [16-18]. These modifications are however more effective before the onset of the diseases and are less effective in the advanced disease states [19]. Prevention and early diagnosis remain the most impactful interventions to reduce the morbidity and mortality due to NCDs especially CVDs [20]. Few studies have evaluated the magnitude and T2D and CVD risk factors among the young adult population, with the conviction that they are at lower risk due to perceived active lifestyles [21,22]. Against such background, this study aimed to investigate the burden of T2D and CVD risk factors (hypertension, dyslipidemia, and abdominal obesity) among a young adult population in an urban setting of Mwanza; with the hypothesis that there is a high magnitude of unattended CVD risk factors with a foreseen danger in NCD morbidity and mortality.

## Methods

**Study design:** this was a community-based cross-sectional study conducted between May and August 2018 in an urban setting of Mwanza city, Tanzania. The study involved young adults aged 18-34 years; males and females, not currently known to be diabetic or hypertensive, and currently resident in Mwanza.

**Sample size estimation:** Kish and Leslie´s formula (1965) was used for the sample size for proportions to estimate the minimum required sample size [23]. Given the global prevalence of 8.8% reported by the International Diabetes Federation (IDF) atlas as global estimates of diabetes in adults aged 20-79 in 2015 and 2040 [24], this study targeted to enrol a minimum of 192 participants.

**Figure 1:**
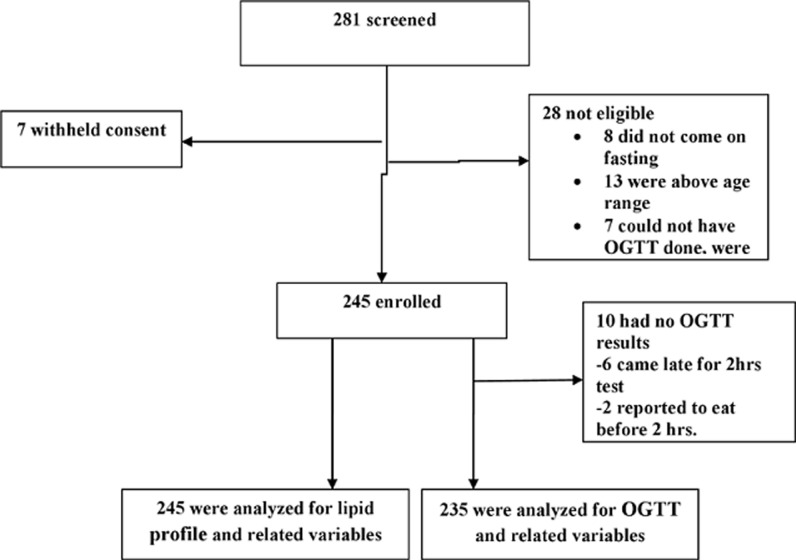
enrolment scheme of the research participants

**Sampling technique:** using multistage random sampling, 245 apparently healthy participants aged 18-34 years were enrolled from 3 representative districts, 2 representing urban wards, and then 4 selected streets [25]. Community leaders were utilized to announce to the public three days before survey day, and all those who turned up at the survey center and met inclusion criteria were enrolled after giving consent (Figure 1).

**Data collection procedures:** all participants were interviewed using structured questionnaires, where social-demographic data were obtained. Finnish Diabetes Risk Score (FINDRISC) questionnaire was administered to all participants and data on age; body mass index (BMI); waist circumference categories; physical activity; vegetable/fruit or berries eating habit; history of hypertension; and hyperglycemia were assessed and documented [26-28]. Weight, height, hip, and waist circumference were measured using a calibrated stadiometer and tape measure under World Health Organization (WHO) protocols [29,30]. Abdominal obesity was defined as waist circumference above 102cm in males and above 88 cm in females [31]. BMI was calculated and categorized under WHO guidelines [29,30].

Blood pressure was measured after 3-5 minutes rest, two times at a space of 5 minutes using a calibrated digital sphygmomanometer (CH-432B, Citizen Systems Japan Co Ltd), and the average value was obtained [32]. Systolic blood pressure of more than 140mmHg and /or diastolic blood pressure of more than 90mmHg were regarded as hypertension [33].

A capillary fingertip blood sample was obtained from each participant for assessment of fasting blood glucose at 0hour and 2hours postprandial blood glucose as part of an oral glucose tolerance test (OGTT), using an ONCALL-PLUS device (ACON Laboratories, Inc. Sandiego, US) [34]. 2-hours blood glucose of more than 11 mmol/l was regarded as diabetes mellitus and levels of 7.8 mmol/l to 11mmol/l as impaired glucose tolerance (IGT) [35]. Five millilitres (5 mL) of fasting venous blood were obtained and serum was harvested for lipid profile analysis. Auto-analyzer machine model ERBA XL (ErbaLachemas.r.o, Brno, Czechia) was used to measure lipid profile including total cholesterol, triglycerides, high-density lipoprotein cholesterol, and low-density lipoprotein cholesterol. Dyslipidemia was defined as levels of total cholesterol of more than 5.2 mmol/L, Low-Density Lipoprotein of more than 3.3 mmol/L, triglycerides of more than 1.7 mmol/L, and High-Density Lipoprotein of less than 1.03 mmol/L in males or less than 1.29 mmol/L in females [36].

Atherogenic indices were calculated from the lipid profile data using the following formula. The atherogenic index of plasma (AIP) is the logarithm of the molar ratio of triglycerides to high-density lipoprotein cholesterol [37,38]. Castelli´s risk index 1 (CRI-I) is the ratio of total cholesterol to high-density lipoprotein cholesterol while Castelli´s risk index 2 (CRI-II) is the ratio of low-density lipoprotein cholesterol to high-density lipoprotein cholesterol [37,39]. Atherogenic coefficient (AC) is the ratio of non-high-density lipoprotein cholesterols to high-density lipoprotein cholesterol [37]. Cholesterol index (CHOLindex) is low-density lipoprotein cholesterol minus high-density lipoprotein cholesterol [37,40,41]. The following were considered the abnormal values of AIP, lipid ratios, and CHOLIndex respectively: AIP >0.1, CRI-I >3.5 in males and >3.0 in females, CRI-II >3.3, AC >3.0, and CHOLIndex >2.07 [40].

**Variables:** the outcome variables were T2D, hypertension, dyslipidemia, and abdominal obesity; while the exposure variables included age, sex, atherogenic indices, level of education, occupation, economic status, physical inactivity, vegetable eating habit, and history of hypertension or diabetes.

**Data management:** all obtained data were double-checked and entered in Excel software and were later exported to STATA IC 13 (Station College, Texas, USA) for cleaning and analysis.

**Data analysis:** continuous variables were checked for normality using the Shapiro-Wilky test. Descriptive statistics were summarized in frequency, means with standard deviation, or median with the inter-quartile range depending on the distribution. Logistic regression analysis was used to assess the associations between dependent variables (dyslipidemia, diabetes, hypertension, abdominal obesity) and independent variables (age, sex, education, occupation, economic status, atherogenic indices). A two-sided p-value of less than 0.05 was considered statistically significant.

**Ethics:** this study obtained an ethical approval from the research and publication committee of Muhimbili University of Health and Allied Sciences (MUHAS) with a Ref number DA_287/298/01.A/. Each participant provided written informed consent before being enrolled in this study. All participants with abnormal results were given health advice and were referred for further health care services.

## Results

**Characteristics of the study participants:** 245 were eligible and recruited in the study; the response rate was 100% for the interviews and other collected data, and 96% (235/245) for the OGTT (Figure 1). The median age of participants was 21 (interquartile range [IQR]: 18 -25) years. The majority were females 148 (60.4%), 192(78.4%) had a higher level of education (college and above). Most participants reported being physically inactive 183 (74.7%) and only 70 (28.6%) reported daily vegetable consumption habits [26]. About 40 (16.3%) of the participants were overweight and 16 (6.5%) were obese based on BMI while 29 (11.8%) of the participants had abdominal obesity (Table 1). About 70% of the participants had an abnormal CRI-I index, while other atherogenic indices were abnormal in 4% to 15% of the study participants (Table 1). Association between diabetes mellitus with participants´ characteristics: Diabetes was present in 7.8% (19) of study participants while 15.5% (38) had IGT (Table 1). There was no observed significant association between diabetes mellitus with age, sex, education, occupation of the study participants, or atherogenic indices (Table 2).

**Table 1:** background characteristics of the study participants

Characteristics	Parameter
**Age in years, Median [Interquartile range (IQR)]**	21(18-25)
**Sex, n (%)**	
**Female**	148(60.4)
**Education level, n (%)**	
Secondary	27(11.0)
Higher	192(78.4)
**Abdominal obesity n (%)**	29(11.8)
**BMI categories, n (%)**	
18-24.99kg/m	165 (67.4)
25-30kg/m	40 (16.3)
Higher than 30kg/m	16 (6.5)
**Personal history of hypertension, n (%)**	
Yes	9 (4.6)
**Personal history of hyperglycemia, n (%)**	
Yes	8(3.3)
**Family history of diabetes, n (%)**	
No	177 (72.2)
Yes, first-degree relative	19 (7.8)
Yes, second degree relative	49 (20.0)
**Glycemic Status (OGTT)**	
IGT n (%)	38 (15.5)
Diabetes n (%)	19 (7.8)
**Hypertension status**	
No hypertension n (%)	161(65.7)
With hypertension (systolic/diastolic) n (%)	84(34.3)
Dyslipidemia - Yes n (%)	111(45.1)
Hypercholesterolemia - Yes n (%)	73 (29.8)
Hypertriglyceridemia - Yes n (%)	26(10.6)
High LDL cholesterol - Yes n (%)	30(12.2)
Low HDL cholesterol- Yes n (%)	32(13.1)
Abnormal AIP n (%)	28(11.4)
Abnormal CRI-I n (%)	185(75.2)
Abnormal CRI-II n (%)	10(4.1)
Abnormal AC n (%)	37(15)
Abnormal CHOLIndex n (%)	17(6.9)

Age has been presented in median with interquartile range. All other parameters are in frequencies with percentages. OGTT: oral glucose tolerance test; IGT: impaired glucose tolerance, LDL: low density lipoprotein cholesterol; HDL: high density lipoprotein cholesterol

**Table 2:** logistic regression of outcome variables with participants’ characteristics

	Outcomes	Diabetes mellitus		Hypertension		Abdominal Obesity		Dyslipidemia	
		Univariate	Multivariate	Univariate	Multivariate	Univariate	Multivariate	Univariate	Multivariate
**Predictors**		OR(95CI)	OR(95CI) P-value	OR (95CI)	OR(95CI)P-value	OR (95CI)	OR(95CI) P-value	OR (95CI)	OR (95CI) P-value
**Age**		1.0 (1-1.1)	1(1-1.1) 0.7	1.1(1-1.1)	1.1(1-1.12) 0.1	1.2(1.1-1.3)	1.3(1.2-1.4) 0.001	1(1-1.1)	1(1-1.1)
**Sex**	Male	1.2(0.7-2.2)	1.2(0.6-2.3)0.3	1.8(1.1-3.1)	2.1(0.9-5.1) 0.09	0.2(0.1-0.6)	0.1(0-0.5)0.007	0.6(0.3-1)	0.6(0.3-1)
**Education level**	Secondary	1.2(0.3-5.1)	1.5(1-2.4) 0.1	0.6(0.2-1.8)	0.9(0.6-1.4) 0.7	0.7(0.2-3.1)	1.8(1-3.3) 0.06	0.8(0.3-2.4)	1.2(0.8-1.7)
	College	1.8(0.6-5.7)		0.7(0.3-1.5)		0.3(0.1-0.9)		1.2(0.5-2.7)	
	Other*	2.6(0.6-11.9)		1.2(0.4-4.1)		3.7(1-14.6)		1.2(0.4-4.1)	
**Occupation**	Self employed	0.9(0.3-2.5)	1.1(0.9-1.4) 0.5	1.8(0.8-4.2)	1.1(0.9-1.4) 0.3	7.1(1.7-29.4)	1.1(0.7-1.7) 0.7	0.6(0.3-1.4)	1(0.8-1.2)
	Salary Employee	1.5(0.6-3.7)		1.6(0.7-3.9)		13.7(3.5-54)		1.1(0.5-2.5)	
	Others*	1.3(0.7-2.6)		1.6(0.9-3.1)		2.3(0.6-9)		0.9(0.5-1.6)	
**Economic status**	Middle	1.1(0.3-4.1)	0.8(0.2-3.5) 1	3.3(0.7-15)	3.9(0.8-19) 0.1	0.8(1.2-3.7)	1.7(0.2-14.3) 0.6	0.8(0.3-2.4)	0.7(0.2-2.3)
**AIP**	Abnormal	1.6(0.7-4) 0.3	1.9(0.7-6.1) 0.3	1.3(0.6-2.9) 0.5	0.9(0.3-2.8) 0.8	1.3(0.4-4) 0.7	0.3(0-2.4) 0.2	-	-
**CRI-I**	Abnormal	1.2(0.6-2.4) 0.7	2(0.6-6.4) 0.2	0.7(0.4-1.2) 0.2	1.4(0.5-3.8) 0.5	10.1(1.4-80.4) 0.02	2.1(0.1-30) 0.6	-	-
**CRI-II**	Abnormal	-	-	1.3(0.4-4.7) 0.7	0.6(0.1-5.1) 0.6	0.8(0.1-6.8) 0.9	0.2(0-4) 0.3	-	-
**AC**	Abnormal	1.3(0.6-3) 0.5	0.8(0.3-2.8) 0.8	1.1(0.5-2.2) 0.9	0.6(0.2-1.8)0.3	3.7(1.6-8.8) 0.003	7.2(1.5-35.1) 0.01	-	-
**CHOL index**	Abnormal	1.2(0.4-4.1) 0.7	3.1(0.5-19.8) 0.2	1.8(0.7-4.8) 0.3	3.1(0.5-19.9) 0.2	3.6(1.2-11) 0.03	1.8(0.2-17) 0.6	-	-

* Others for education included university level or higher. Others (for occupation) included casual works, paid on daily or weekly basis. References for sex (female), Education level (primary education) Occupation (not employed), Economic status (low) and for AIP, CRI-1, CRI-II, AC and CHOLIndex are their normal values

**Association between hypertension with participants´ characteristics:** more than one-third of the study participants had systolic/diastolic hypertension. Isolated diastolic hypertension was more prevalent than isolated systolic hypertension (Table 1). In a univariable model, the risk of hypertension increased with age (OR 1.1, 95%CI 1.0-1.1) and with male sex (OR 1.8, 95%CI 1.1-3.1). However, in a multivariable model, there was no observed significant association (Table 2). Association between dyslipidemia with Participants´ characteristics: forty-one percent (41.2%) of the study participants had dyslipidemia. High cholesterol levels and high triglyceride levels were more prevalent than other lipid profile derangements (Table 1). The female gender increased risk for dyslipidemia about twice compared to males (OR 1.8, 95%CI 1-3) (Table 2).

**Association between abdominal obesity with participants´ characteristics:** abdominal obesity was present in 11.84% of the study participants (Table 1). Females had 12 times increased risk of abdominal obesity than males, (OR 12.4, 95%CI 3.3-46.7). Increased age significantly increased the risk of abdominal obesity (OR 1.3, 95%CI 1.2-1.4), the atherogenic coefficient was significantly associated with abdominal obesity (OR 7.2, 95%CI 1.5-35.1) (Table 2).

## Discussion

Here we report high levels of DM, hypertension, dyslipidemia, and abdominal obesity among young people in an urban setting in Tanzania. Our findings also show that a low level of physical activity is a major modifiable risk factor associated with the occurrence of these diseases. With demography dominated by a relatively younger population in LMICs, understanding the magnitude of major NCDs and related factors in young adults is paramount. These findings underscore the need for strengthening interventions that target cardio-metabolic risk factors in a young population.

Recent studies have also shown a high prevalence of diabetes in young adults under 40 years of age [13,42]. However, the observed prevalence of diabetes mellitus (7.8%) in this study is higher compared to the recently reported prevalence of diabetes in urban Tanzania of 3.2 - 6.9% for the age group of 20-34 years [24]. This value approaches the estimated prevalence of 9% projected for the year 2030 [43]. This trend is alarming and indicates the possible danger that Tanzanian young adults are exposed to in the absence of active interventions. It has been reported that cardiovascular diseases develop more rapidly in those with IGT starting at an early age than those whom it starts late in life [42]. The lifestyle of college students, including the consumption of food that is rich in fats, is not healthy and should be addressed to combat this rise. More studies should be done in other settings, including rural areas to have enough data on the burden of diabetes in young adults, and also to probe potential risk factors and possible preventive measures.

An alarming prevalence of hypertension has been observed in this study. This is supported by recent studies in the same age group by Nsanya *et al*. 2019, where the combined prevalence of high blood pressure in young adults and adolescents of Tanzania and Uganda was 40%, showing the seriousness of this problem in the population [44]. Data from studies done among school children and adolescents in Tanzania and other low-income countries have also reported a significantly higher prevalence of hypertension [45,46]. However, data from developed countries suggest a much lower prevalence of hypertension in this age group [47]. We hypothesize that hypertension among young adults in urban settings in Africa is contributed by several factors such as a change of ordinary to a sedentary lifestyle which is on the rise, dietary factors that are yet to be studied, exposure to free radicals, and intake of fewer antioxidants. This hypothesis needs to be studied for better understanding of these differences. Isolated diastolic hypertension was found to be more prevalent in our study subjects. This finding is in keeping with other studies that described diastolic hypertension to be common in younger ages [48] and has been associated with the onset of systolic hypertension as well as the development of systolic/diastolic hypertension and other cardiovascular complications [48]. It has also been found that diastolic pressure and diastolic hypertension drive coronary risk in younger subjects, so it´s paramount to intervene in this trend in the young population in this region [49]. The prevalence of dyslipidemia in this study is far higher (41.22%) than what was reported in the Bogalusa heart study in 2003, where the prevalence of dyslipidemia in blacks was found to be 4.7% [50]. The prevalence of dyslipidemia among young adults in India was reported to be as high as 36% [9]. This is alarming as free fatty acids and adipokines can impair insulin sensitivity and bring about the cascade of insulin resistance, hyperinsulinemia, and T2D [51]. Some studies have observed that free fatty acids contribute to obesity-associated insulin resistance and later development of diabetes via up-regulation of microRNA 21 (miR-21) [52]. Dyslipidemia is, therefore, an important factor in the development of T2D and its control is of paramount importance in the fight against early onset of T2D in a young population. Although most of the atherogenic indices studied were abnormal in up to 15% of the participants, only one index (atherogenic coefficient), was associated with abdominal obesity. There were no other observed associations between atherogenic indices with the currently studied diseases. Nonetheless, since the atherogenic indices have been shown to best predict future cardiovascular diseases [37,38], it is concerning that younger population in African settings are at increased risk which further cements the need for early interventions.

We observed a higher prevalence of abdominal obesity (11.84%) in the current study. With this finding, we foresee higher rates of cardiovascular diseases shortly if the situation is left unchecked. This rate is similar to the recently reported data from Dodoma Tanzania, where the prevalence of abdominal obesity among adults aged 18-30 years was 12.96% and was higher in people with a higher level of education and who were physically inactive [15]. The study also shows that the majority of study participants were physically inactive [26]. This finding is in agreement with reports from other studies which showed a high prevalence of abdominal obesity [14,15,53], it is high time to employ intervention towards the rise and rescue the future. It is well-known that obesity accounts for more than 80% of the pathophysiology of metabolic syndrome [51], weight loss is, therefore, an important step in reducing the incidence of these disorders, especially in a younger population.

Despite these findings, our study had limitations. Participants in this study were college and university students, compared with the general community. Because of the differences in lifestyle between college and university students compared to the general community, it is possible that the observations made may not be representative of the status of metabolic disorders in the general population of young adults. Moreover, we did not assess other lifestyle risk factors, including the use of anti-glycemic medication which is not included in the FINDRISK questionnaire, which could have contributed to these findings. We were also not able to test and differentiate the various forms of diabetes mellitus, and may therefore have included individuals with type 1 diabetes (T1D) which is not associated with lifestyle risk factors but could be found in this younger population, although previous studies have indicated the prevalence of T1D of less than 1% in African population [54].

**Strength and limitations:** this study has explored the hidden burden of diabetes mellitus, hypertension, dyslipidemia, and abdominal obesity in the young adult population, a population that is considered to have an active lifestyle and is seldom studied. Despite the relatively smaller sample size and single-centered nature of this study which may limit the generalization of its findings, this study is an eye-opener to the hidden risk of CVD among young adults which calls for larger studies to strengthen the evidence.

## Conclusion

The high magnitude of T2D, dyslipidemia, hypertension and abdominal obesity was observed among the young adult population in Urban Mwanza, Tanzania. The high prevalence of CVD risk factors in this population hints toward a significant burden of CVDs in the near future.

### What is known about this topic

- There is a rise of non-communicable diseases in low and middle-income countries; more pronounced in perceived high-risk groups such as older ages and people with other co-morbidities such as HIV.

### What this study adds

- The current study reveals the hidden threat; the burden of cardiovascular risk factors in a young population which is not perceived to be at risk and therefore receiving less attention.
